# Revisiting potential value of antitumor drugs in the treatment of COVID-19

**DOI:** 10.1186/s13578-022-00899-z

**Published:** 2022-10-01

**Authors:** Wenfang Zheng, Zekun Zeng, Shumei Lin, Peng Hou

**Affiliations:** 1 Department of Endocrinology, The First Affiliated Hospital of Xi’an Jiaotong University, Xi’an, 710061 People’s Republic of China; 2 Department of Infectious Diseases, The First Affiliated Hospital of Xi’an Jiaotong University, Xi’an, 710061 People’s Republic of China; 3 Key Laboratory for Tumor Precision Medicine of Shaanxi Province, The First Affiliated Hospital of Xi’an Jiaotong University, Xi’an, 710061 People’s Republic of China

**Keywords:** COVID-19, Antitumor drugs, SARS-CoV-2, Drug repurposing, Cytokine storm

## Abstract

Since an outbreak started in China in 2019, coronavirus disease 2019 (COVID-19) has rapidly become a worldwide epidemic with high contagiousness and caused mass mortalities of infected cases around the world. Currently, available treatments for COVID-19, including supportive care, respiratory support and antiviral therapy, have shown limited efficacy. Thus, more effective therapeutic modalities are highly warranted. Drug repurposing, as an efficient strategy to explore a potential broader scope of the application of approved drugs beyond their original indications, accelerates the process of discovering safe and effective agents for a given disease. Since the outbreak of COVID-19 pandemic, drug repurposing strategy has been widely used to discover potential antiviral agents, and some of these drugs have advanced into clinical trials. Antitumor drugs compromise a vast variety of compounds and exhibit extensive mechanism of action, showing promising properties in drug repurposing. In this review, we revisit the potential value of antitumor drugs in the treatment of COVID-19 and systematically discuss their possible underlying mechanisms of the antiviral actions.

## Background

The unexpected pandemic of coronavirus disease 2019 (COVID-19) caused by infection with severe acute respiratory syndrome coronavirus 2 (SARS-CoV-2), has swept the globe and inevitably causes serious global health crisis. Currently, the clinical treatment for COVID-19 is primarily supportive care. Several investigational drugs which have shown promising antiviral activities or immunomodulatory effects are under evaluation in clinical trials for COVID-19, and some have progressed to clinical use. Remdesivir is a broad-spectrum adenosine analogue antiviral drug which interferes with viral replication via inhibition of RNA-dependent RNA polymerase (RdRp) [1]. It received Food and Drug Administration (FDA) approval on October 22, 2020, as an antiviral drug against COVID-19 [2]. Besides, other antiviral drugs including protease inhibitors and interferon-α are still being tested in preclinical or clinical trials. However, the available data indicate that the therapeutic effects of those drugs including remdesivir on COVID-19, are less notable. Thus, there is an urgent need to find more effective medications for COVID-19, which can be achieved by developing new agents or repurposing existing drugs, the latter also referred to as drug repurposing.

Drug repurposing is a strategy to explore novel and broader applications for approved drugs or investigational agents beyond their original therapeutic indications [3]. This strategy presents multiple advantages over developing new drugs, especially for emerging diseases. The development of a new drug is time-intensive and costly, and is often accompanied by high risk of failure [4]. Thus, it can hardly satisfy the pressing medical need for an emerging disease. Drug repurposing, in contrast, is a time-saving strategy with lower cost, thereby providing a faster way to discover potential drugs that already have safety profiles and favorable pharmacological properties [5]. With favorable safety profiles, these repurposed drugs can quickly enter into clinical trials and being tested, which is more likely to meet the urgent medical demands for emerging diseases. Moreover, repurposed agents may reveal novel targets and molecular pathways that can be further studied in emerging diseases. Altogether, these advantages make drug repurposing a much more valuable strategy to identify potential therapeutic drugs for emerging diseases, especially for some emerging infectious diseases lacking of effective treatments. At present, there are many successful examples of drug repurposing. Thalidomide, a synthetic derivative of glutamic acid, was originally prescribed as a sedative and antiemetic for morning sickness, but was withdrawn due to severe teratogenicity in multiple tissues and organs of infants [6]. However, it was later found to be effective in patients with erythema nodosum leprosy and multiple myeloma, and was approved by FDA in 1998 and 2006, respectively [7–9]. Once abandoned due to severe adverse effects, thalidomide demonstrated its therapeutic value in other diseases and achieved substantial commercial success. Another example is chlorcyclizine, a first-generation antihistamine widely used for allergic diseases, was shown to have high antiviral activity against hepatitis C virus (HCV) in vitro [10]. And in vivo studies further demonstrated its efficacy in inhibiting genotypes 1b and 2a HCV infection [10]. In addition, the results of a randomized proof-of-concept clinical trial showed that chlorcyclizine had some anti-HCV effects, and most notably in combination with ribavirin, a broad-spectrum antiviral agent [11].

In the last decades, tremendous efforts have been made to elucidate the molecular mechanisms involved in cancer and develop therapeutic approaches. Numerous anticancer drugs have been developed, contributing to improved prognostication of cancer patients. In addition to antitumor effects, many drugs also display other pharmacological actions, which indicates extended indications beyond cancer. For example, some anticancer drugs like methotrexate are widely used to treat autoimmune disorders. In recent years, researchers have sought to explore the therapeutic role of antitumor drugs in COVID-19, and some drugs stand out for their potential therapeutic effects. Further mechanistic studies have demonstrated those potential drugs exert their therapeutic impacts via directly disturbing the virus life cycle or indirectly modulating the host immune responses. In this review, the potential therapeutic role of antitumor drugs in COVID-19 and their underlying mechanisms will be systematically discussed.

## Common pathophysiological features between COVID-19 and cancer

SARS-CoV-2 is an enveloped RNA virus with a single-stranded positive-sense RNA, belonging to the family Coronaviridae [12]. It contains four main structural proteins, envelope (E), membrane (M), nucleocapsid (N) and spike (S) proteins [13]. The S protein consists of two distinct subunits S1 and S2, whose primary role is facilitating membrane fusion thus enabling SARS-CoV-2 to enter susceptible cells. The S1 subunit can directly interact with human angiotensin-converting enzyme 2 (ACE2) through a receptor-binding domain (RBD) [14]. The S2 subunit encloses a fusion peptide which prompts membrane fusion upon infection of target cells [15]. Cellular proteases play an important role in viral entry process, because S1 and S2 are noncovalently bound before fusion and S protein priming is required to expose fusion peptide that catalyzes fusion [16]. Current studies demonstrate that the cleavage of S protein can be mediated either by type 2 transmembrane serine protease (TMPRSS2) on the surface of infected cell or by cathepsin L in the endosomes following endocytosis [15]. TMPRSS2 is a transmembrane protease and is frequently employed by respiratory viruses including severe acute respiratory syndrome coronavirus (SARS-CoV) to activate their fusion proteins [17]. Cathepsin L is a cysteine protease and has been found to activate S glycoprotein through proteolysis within endosomes upon SARS-CoV infection [18]. Evidently, SARS-CoV-2 seems to preferentially rely on TMPRSS2 for S protein activation over cathepsin L [19]. ACE2 is expressed at low levels in multiple human tissues including airways, cornea, colon, liver, heart, kidney, etc., while TMPRSS2 displays a broader expression profile [20]. Nonetheless, they have a common feature, which are primarily expressed in epithelial cells including type II pneumocytes [20, 21].

Once the virus successfully enters the cell, the genomic RNA is freed to acts as messenger RNA (mRNA) to synthesize two polyproteins pp1a and pp1ab which further generate nonstructural proteins (nsps) through proteolysis. The proteolysis process is mediated by two viral proteases: the main protease (Mpro, also termed 3CLpro) and the papain-like protease (PLpro) [22]. The nsps then form replicase-transcriptase complex to transcribe the viral genomic and sub-genomic RNA, followed by the translation of structural proteins. Finally, complete viral particles are successfully assembly and released into the extracellular space.

It is the fact that COVID-19 and tumor share many common pathophysiological features. Firstly, both of them are associated with dysregulated immune inflammatory responses. In response to SARS-CoV-2 invasion, the host antiviral immunity including innate and adaptive immune response is mobilized to eliminate the virus. The innate immune system serves as the first line of host defense against viral infection. However, diminished and delayed innate immune response has been observed in those with moderate to severe COVID-19, which allows SARS-CoV-2 to evade immune surveillance and spread in the early stage of infection [23]. Overzealous immune response in a later stage manifesting as hyperactivation of immune cells and “cytokine storm” characterized by a surge of blood cytokine levels may lead to local or systemic tissue damage or, more seriously, multiorgan dysfunction [24]. Indeed, a number of patients with COVID-19 display varying degrees of alveolar damage and interstitial mononuclear inflammatory infiltrates [25]. Meanwhile, the increase of type 17 helper T (Th17) cells and high cytotoxicity of CD8 T cells as well as elevated circulating pro-inflammatory cytokines, such as interleukin-6 (IL-6), tumor necrosis factor, interferon-γ (IFN-γ) and granulocyte–macrophage colony stimulating factor (GM-CSF), in peripheral blood of the patients may partly account for the immune injury [24–26]. With respect to cancer, host immunity and inflammation have long been recognized as important players in the initiation and progression of cancer. Despite the high plasticity of the components of the innate immune system, tumor-inhibiting or tumor-promoting, which is mostly context dependent, the antitumor potential of some innate immune cells can be leveraged and expanded to develop immunotherapies against cancer [27]. Some antitumor drugs have been developed to boost the innate immunity, which may be repurposed as antiviral agents to induce and amplify the innate antiviral response in patients with COVID-19. Tumor-promoting inflammation is considered as an enabling characteristic for its pivotal role in facilitating the acquisition of core hallmark capabilities [28]. Tumor cells can recruit inflammatory cells and regulatory immune cells by secreting proinflammatory cytokines to compromise antitumor immunity, thereby evading immune cell elimination. Accordingly, targeting proinflammatory factors is a promising antitumor strategy which enhances antitumor immunity by modulating immune status of the tumor environment.

Secondly, SARS-CoV-2 infected cells exhibit anomalous metabolism which is also a hallmark of cancer cells. Once inside the cell, SARS-CoV-2 will hijack host cell metabolic pathways to bolster self-replication. Besides, SARS-CoV-2 can remodel metabolic patterns of host cell to create a hospitable cellular environment favoring viral replication. Lipid metabolism is the most frequently altered metabolic pathway upon SARS-CoV-2 infection, which is involved in post-translational modification of certain proteins critical for viral entry and replication [29]. Likewise, metabolism reprogramming is also considered as a prominent hallmark of tumor cells [30]. In addition to enhanced anaerobic glycolysis, lipid metabolism reprogramming is also a significant metabolic alteration in cancer and is intricately related to malignant phenotypes of tumor cells [31, 32]. Aside from lipid metabolism, both SARS-CoV-2 and tumor cells have a vigorous demand of purines and pyrimidines for nucleic acid synthesis, which acts as replication stress driving metabolic alterations of host cells or tumor cells [33]. Overall, these adaptive changes of metabolism process are in favor of viral replication and tumor development. Thus, targeting aberrant metabolic pathways is a reasonably therapeutic strategy. Similar alterations of cellular metabolism induced by either external factors such as SARS-CoV-2 infection or intrinsic factors like tumorigenesis lay a theoretical foundation for repurposing antitumor drugs in treating COVID-19.

## Therapeutic potential of antitumor drugs for COVID-19

**Fig. 1:**
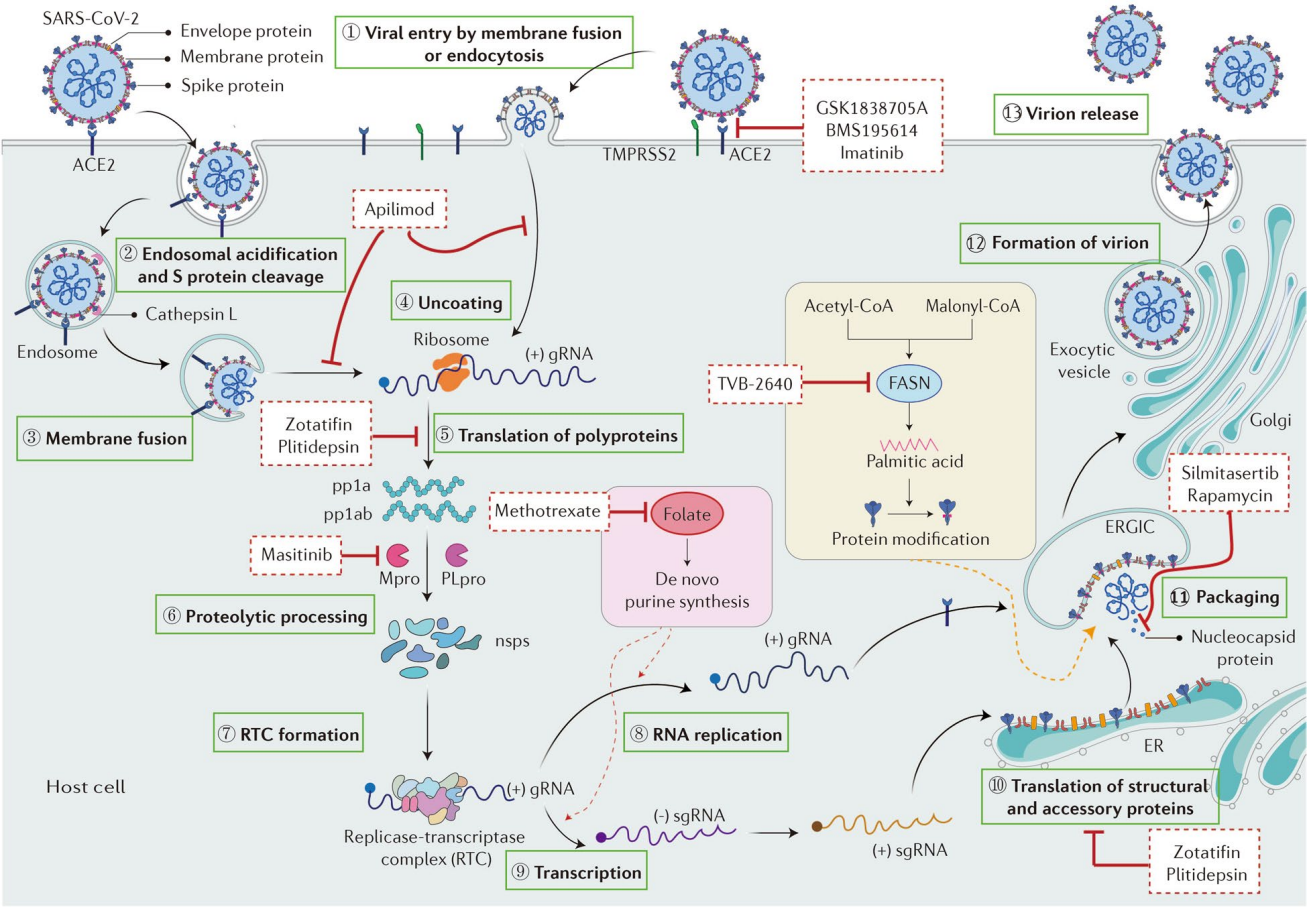
Schematic representation of SARS-CoV-2 life cycle within host cells and potential antitumor drugs to treat COVID-19. The entry of SARS-CoV-2 into target cells requires fusion of viral and cellular membrane, which is initiated by conformational change of spike (S) protein. The S protein of SARS-CoV-2 binds to angiotensin- converting enzyme 2 (ACE2) on the host cells. If the host cell has low expression of type 2 transmembrane serine protease (TMPRSS2), the virus is internalized by endocytosis and transported to endolysosomes where S protein is processed by the protease cathepsin L (step 2) and then followed by membrane fusion (step 3). In the presence of TMPRSS2, it can directly induce the cleavage of S protein on the cell surface and trigger membrane fusion. Once SARS-CoV-2 enters into the cell, viral RNA is released into the cytoplasm (step 4) and is translated to two polyproteins, pp1a and pp1b (step 5), which are proteolytically cleaved into non-structural proteins (nsps) (step6). Then nsps form replicase-transcriptase complex (RTC) (step 7), which transcribes the viral genomic and subgenomic RNA (sgRNA) (steps 8 and 9). The sgRNAs are translated to produce structural and accessory proteins (step 10). Subsequently, nucleocapsids are assembled and bud into the lumen of the endoplasmic reticulum (ER)-Golgi intermediate compartment (ERGIC) (step 11) to produce a complete virus particle. Finally, virions traffic to the Golgi (step 12) and exit the host cell (step 13). Some antitumor drugs can target different stage of the viral cycle thus exerting antiviral effects. (Created with the assistance of smart.servier.com)

Dysregulated host immune responses and aberrant host metabolism are observed as common characteristics of COVID-19 and cancer, signifying the possibility of repurposing antitumor agents for the treatment of COVID-19. Mechanistically, those antitumor drugs with potential antiviral properties can be split into two categories. The first interferes with viral entry, replication and/or assembly to break off the life cycle of SARS-CoV-2 (Fig. 1). The second category encompasses those antitumor drugs endowed with immunoregulatory activities which may boost innate immunity or mitigate excessive inflammatory responses in severe COVID-19 cases. A complete list of potential drugs along with their targets, mechanism of action and embroiled clinical trials are shown in Table 1.

**Table 1:** Summary of potential anticancer drugs in the treatment of COVID-19

Class	Mode of action	Target	Agent	Clinical trial	Refs
Inhibition of viral entry	Inhibition of viral entry and endosome trafficking	PIKfyve	Apilimod	NCT04446377	[41]
	Allosterically inhibiting the binding of ACE2 to S protein	ACE2	Imatinib	NCT04394416 NCT04953052 NCT04794088	[52]
Interference with the viral cycle	Inhibition of protein translation	eIF4A	Zotatifin	NCT04632381	[56]
		eEF1A2	Plitidepsin	NCT04382066 NCT04784559	[59]
	Blockade of viral replication	FASN	TVB-2640	NA	[66]
		Folate metabolism	Methotrexate	NCT04610567	[33]
		CK2	Silmitasertib	NCT04663737 NCT04668209	[76]
		mTORC1	Rapamycin	NCT04341675 NCT04948203 NCT04461340	[82]
	Competitive inhibition of Mpro	Mpro	Masitinib	NCT05047783	[84]
Inhibition of overactivated inflammatory responses	Blockade of excessive cytokines	IL-6	Tocilizumab	NCT04356937 NCT04320615 NCT04372186 NCT02735707	[108, 109]
		JAK	Tofacitinib	NCT04750317 NCT04469114	[117]
			Baricitinib	NCT04358614 NCT04421027 NCT04362943 NCT04401579 NCT04640168	[118]
			Ruxolitinib	NCT04334044 NCT04362137 NCT04338958 NCT04374149 NCT04359290	[119]
		TNF-α	Thalidomide	NCT04273581 NCT04273529	[24]
			Lenalidomide	NCT04361643	[24]
		TOP1	Topotecan	NCT05083000	[131]
	Activation of innate immune response	STING	diABZI	NA	[97]
	Inhibition of the production of active neutrophil	Neutrophils	Corticosteroids	NCT04530409 NCT04726098 NCT04355637 NCT05004753	[124]
	Inhibition of the production of proinflammatory cytokines from immune cells	BTK	Ibrutinib	NCT04375397 NCT04439006 NCT04848493	[138]
			Acalabrutinib	NCT04647669 NCT04497948 NCT04380688 NCT04665115	[139]
			Zanubrutinib	NCT04382586	[139]
	Inhibition of angiogenesis	VEGF	Bevacizumab	NCT04275414 NCT04822818	[128]

S protein Spike proteins; *PIKfyve* Phosphatidylinositol 3-phosphate 5-kinase; *ACE2* Angiotensin-converting enzyme 2; *eIF4A* Eukaryotic translation initiation factor 4A; *eEF1A2* Eukaryotic translation elongation factor 1 alpha 2; *FASN* Fatty acid synthase; *CK2* Casein kinase II; *mTORC1* Mammalian target of rapamycin complex 1; *Mpro* The main protease; *IL-6* interleukin-6; *JAK* Janus kinase; *TNF-α* Tumor necrosis factor-alpha; *TOP1* Topoisomerase 1; *STING* Signaling effector stimulator of interferon genes; *diABZI* Dimeric amidobenzimidazole; *BTK* Bruton’s tyrosine kinase; *VEGF* Vascular endothelial growth factor

## Drugs interfering with the entry of SARS-CoV-2 into susceptible cells

The interactions between S protein and host cell receptor ACE2 facilitate SARS-CoV-2 entry. Disrupting this process is thus a way to intercept viral invasion. A previous study identified drug candidates that were capable of hindering the interactions between S protein and ACE2 by a high-throughput virtual screening approach [34]. Two anticancer drugs, GSK1838705A and BMS195614, were found to target RBD of S protein (S-RBD) [34]. GSK1838705A is a highly potent and reversible kinase inhibitor of insulin-like growth factor-1 (IGF1) receptor (IGF1R), showing antitumor activity in several solid tumors and hematological malignancies [35, 36]. In the structure-based screening, GSK1838705A displayed binding energies of -6.46 kcal/mol with S-RBD [34]. BMS195614 is a selective antagonist of retinoic acid receptor alpha (RARα) [37]. Retinoic acid receptors (RARs) are ligand-dependent transcription factors that act as heterodimers with retinoid X receptors (RXRs) to participate in the regulation of organogenesis, organ homeostasis, cell proliferation and differentiation [38]. RARα antagonists have been demonstrated to suppress tumor growth both in vitro and in vivo [39, 40]. BMS195614 showed a relatively large binding energy of − 8.25 kcal/mol with S-RBD residues [34]. Taken together, GSK1838705A and BMS195614 are potential inhibitors of S-RBD; however, further studies are needed to validate their efficacy in preventing SARS-CoV-2 infection.

A recent study identified potential drug candidates for COVID-19 by a high-throughput re-profiling screen with existing clinical-stage or approved agents. Among them, apilimod showed the highest antiviral activity against SARS-CoV-2 in vitro and ex vivo. Further studies indicated that apilimod acted on viral entry phase of SARS-CoV-2 life cycle [41]. Apilimod is a highly selective phosphatidylinositol 3-phosphate 5-kinase (PIKfyve) inhibitor and establishes robust cytotoxic effect against B-cell non-Hodgkin lymphoma [42]. A phase 1 clinical trial to appraise the safety and pharmacokinetics of apilimod in patients with non-Hodgkin lymphoma was recently completed (NCT02594384). PIKfyve is a FYVE finger-containing phosphoinositide kinase that primarily phosphorylates phosphatidylinositol 3-phosphate (PI3P) to phosphatidylinositol 3,5-bisphosphate (PI(3,5)P2), one of the phosphoinositides involving in the regulation of endosome dynamics [43]. The endosome system functions as a sorting machine to deal with the materials internalized by endocytosis and participates in various cellular process including endocytosis, biogenesis of organelles, metabolism and regulation of multiple signal transduction pathways [44]. Dysregulation of endosome pathways has been found to be involved in cancers, neurodegenerative diseases, inflammatory disorders, infectious diseases, etc. [42, 45]. As a critical enzyme contributing to PI(3,5)P2 production, PIKfyve is an important regulator of endosomal pathway [45]. The cytotoxic effect of apilimod on B-cell non-Hodgkin lymphoma is mainly mediated by PIKfyve inhibition which disrupts endosomal and lysosomal homeostasis, thereby leading to cellular dysfunction and cancer cell growth arrest [42]. However, the mechanism over which apilimod exerts its inhibitory effect on SARS-CoV-2 remains largely unknown.

To date, there are at least two proposed pathways for SARS-CoV-2 entry into cells—by direct fusion of the virus with host cell membrane or by receptor-mediated endocytosis. There is evidence demonstrating that apilimod interrupts normal endosome trafficking by inhibiting PIKfyve activity, and in such a way it thwarts SARS-CoV-2 invasion [46]. This is further supported by the imaging data which reveal accumulation of virus particles in vacuolated endosomes and the trapped virions are unable to be released into the cytosol [46]. And the study also shows that apilimod may interfere with the maturation of cathepsin without affecting its activity, although other studies report that apilimod reduces cathepsin activity [46, 47]. Despite the discrepancy between different studies, apilimod is being tested in a phase 2 clinical trials for COVID-19 (NCT04446377). Thus, more efforts are still needed to unravel the molecular mechanisms underlying apilimod-mediated inhibition of SARS-CoV-2 infection for a better understanding of host-virus interaction during viral infection.

As a systemic disease, COVID-19 may provoke serious metabolic complications with multiple organ systems involvement, including endocrine system, cardiovascular system, immune system, etc. [48–50]. However, the underlying mechanism of metabolic defects induced by SARS-CoV-2 infection is not well understood. ACE2 is known to be a receptor of SARS-CoV-2 and inhibition of it has been suggested as a promising strategy for COVID-19 treatment [51]. However, a recent study has unveiled a novel role of ACE2 in maintaining systemic metabolic homeostasis beyond its role in viral entry [52]. Notably, this study demonstrated that impaired ACE2 pathway might be a key player in SARS-CoV-2 related metabolic disorders, and identified imatinib, methazolamide and harpagoside as direct enzymatic activators of ACE2 by a high-throughput compound screening [52]. In insulin-resistant mice infected with SARS-CoV-2, treatment with imatinib restored the enzymatic activity of ACE2 and ameliorated the defects in glucose and lipid metabolism [52]. Imatinib, an orally bioavailable tyrosine kinase inhibitor, is used clinically to treat chronic myeloid leukemia and gastrointestinal stromal tumors [53]. In fact, imatinib has been previously identified as a potential inhibitor of SARS-CoV-2 entry; however, the detailed mechanism is unclear [54]. As supported, a previous study demonstrated that imatinib blocked the access of SARS-CoV-2 to host cells by allosterically inhibiting the binding of ACE2 to S protein [52]. Encouragingly, these findings redefine the role of ACE2 in SARS-CoV-2 infection and confer imatinib new identity as an antiviral agent. Currently, several clinical trials are in progress to evaluate the potency of imatinib in hospitalized adults with COVID-19.

## Drugs interfering with SARS-CoV-2 replication, assembly and release

SARS-CoV-2 can hijack and remodel host cell biological pathways including translation, nucleic acid metabolism, protein homeostasis, folate and carbon metabolism and energy metabolism, thereby creating an environment conducive to its replication [55]. Due to the dependency of virus on host cells, targeting host cell pathways is an alternative antiviral approach which indirectly disturbs SARS-CoV-2 replication, assembly and release. Based on this, many studies have been carried out to identify host factors obligatory for SARS-CoV-2 infection.

### Targeting host translational machinery

Like all coronaviruses, SARS-CoV-2 relies heavily on host cap-dependent translation to synthesize viral proteins [56]. The protein synthesis inhibitors cycloheximide and emetine are able to inhibit SARS-CoV-2 replication in vitro [55]. Interestingly, some antitumor drugs also exhibit the capacity to repress the translation process in host cells. Zotatifin (eFT226), a selective eukaryotic translation initiation factor 4A (eIF4A) inhibitor, promotes the binding of eIF4A to specific polypurine sequence motifs in the 5’-UTR of zotatifin target genes and stalls the scanning of 43S pre-initiation complex along the mRNA, leading to suppression of translation initiation [57]. The expression of some oncogenic drivers containing polypurine motifs is regulated by the activity of eIF4A. Recently, several studies indicated that zotatifin inhibited tumor cell proliferation and induced cell apoptosis by modulating phosphatidylinositol 3-kinase/protein kinase B/mammalian target of rapamycin (PI3K/AKT/mTOR) pathway [57, 58]. Moreover, a clinical trial (NCT04092673) is currently underway to assess antitumor efficacy of zotatifin in patients with advanced solid tumor malignancies. Owing to the important role of eIF4A in protein synthesis, zotatifin displays robust antiviral activity in vitro (90% inhibitory concentration (IC_90_) = 0.037 μM) by inhibiting the biogenesis of N protein which is indispensable for SARS-CoV-2 replication and assembly [56]. Meanwhile, the assessment of its safety and antiviral activity is ongoing in a phase 1 clinical trial (NCT04632381).

Another antitumor drug, plitidepsin (aplidin), also exhibits potent antiviral activity against SARS-CoV-2 in preclinical studies [59]. It is an inhibitor of eukaryotic translation elongation factor 1 alpha 2 (eEF1A2) and is used clinically to treat multiple myeloma [60]. Plitidepsin was found to hold powerful anti-SARS-CoV-2 property in several cell-based assays elicited from suppressing the expression of N protein [59]. And the infected cells showed reduced genomic RNA content and subgenomic RNA expression in the presence of plitidepsin [59]. Surprisingly, plitidepsin-mediated inhibitory effects were more potent and longer lasting than remdesivir [59]. In this study, two different mouse models of SARS-CoV-2 infection were established to evaluate the in vivo efficacy of plitidepsin. The results showed that plitidepsin-treated mice exhibited reduced viral lung titers and lung inflammatory pathology over remdesivir-treated mice [59]. Taken together, both zotatifin and plitidepsin show great potential in treating COVID-19. A multicenter randomized proof-of-concept clinical trial for safety evaluation of plitidepsin was completed (NCT04382066), demonstrating a favorable safety profile of plitidepsin as well as a therapeutic benefit for patients with COVID-19 [61]. Besides, a phase 3 clinical trial is ongoing to further assess the safety and efficacy of it (NCT04784559).

### Targeting host metabolic process

As obligatory intracellular parasites, viruses are entirely dependent on host cell metabolism to obtain raw materials for biological synthesis. After entry into the cell, viruses exploit and modulate host cell metabolic machinery to create a cellular environment favorable for viral replication and spreading. Blocking those virus-favorable metabolic pathways is a valuable approach to impede viral replication. In fact, the connection between virus and host cell metabolism has been investigated in a variety of viruses including human immunodeficiency virus (HIV), the Zika virus (ZIKV) and HCV [62–65]. As for COVID-19, there are studies showing that SARS-CoV-2 infection instigates multiple metabolic alterations of host cell to meet the demands of replication: (1) SARS-CoV-2 exploits fatty acid synthesis pathway for structural proteins palmitoylation and virion assembly [66]; (2) SARS-CoV-2 hijacks folate and one-carbon metabolism to generate intermediates for de novo purine synthesis [33]; (3) SARS-CoV-2 activates the de novo pyrimidine synthesis pathway to favor viral replication and evade antiviral immune response [67].

Increased de novo fatty acid synthesis has been observed as a common event in human cancers [68]. Overexpression of lipogenic enzymes especially fatty acid synthase (FASN), the key enzyme in fatty acid synthesis, is common in multiple cancers [69]. Accumulated evidences indicate that FASN inhibition can kill cancer cells through diverse mechanisms, including disrupting lipid membrane structure, reducing protein palmitoylation and perturbing oncogenic signaling pathways FASN involved in [70]. Substantial efforts have been made to develop FASN inhibitors and many of them, such as cerulenin, GSK837149A and orlistat, have shown antitumor properties in preclinical studies [71–73]. However, none of them have been tested in cancer patients due to their poor pharmacologic properties or extensive side effects. Later, the emergence of new generation FASN inhibitors brings new hopes to this dilemma. TVB-2640, a highly selective and reversible FASN inhibitor, has successfully completed phase 1 clinical trials (NCT02223247) in patients with solid tumors and demonstrates a favorable tolerability profile when administered orally [74]. Recent studies have identified several FASN inhibitors as drug candidates for COVID-19, including TVB-2640 [66]. TVB-2640 significantly inhibited SARS-CoV-2 infection [half maximal effective concentration (EC_50_) = 4 nM] and replication in vitro [66]. In a mouse model of SARS-CoV-2 infection, intraperitoneal administration of TVB-2640 (8 mg kg^−1^ body weight) attenuated body weight loss and extended lifespan of SARS-CoV-2-infected mice [66]. Notably, TVB-2640 showed similar inhibitory effects on the new SARS-CoV-2 variants in vitro [66]. These results strongly imply the important role of lipid synthesis pathway in SARS-CoV-2 replication and its potential value as a therapeutic target for COVID-19.

Folate metabolism participates in diverse physiological processes including nucleic acid synthesis, amino acid homeostasis, epigenic regulation and cellular redox homeostasis, which is critical for both normal cells and tumor cells [75]. Consistent with the high proliferative capacity of tumor cells, one-carbon metabolic pathway remains highly active in cancer cells for the biosynthesis of nucleotides. Thus, targeting folate-mediated one-carbon metabolism is an effective way to combat cancer. Two known chemotherapeutic agents, methotrexate and 5-fluorouracil, targeting one-carbon metabolic pathways, have already been used for the clinical treatment of many neoplastic diseases. Given that SARS-CoV-2 relies on host cellular metabolism for the acquisition of raw materials for replication, there was a study attempting to explore the role of host folate and one-carbon metabolism in viral replication by performing transcriptional and metabolomic analyses [33]. The results showed that folate and one-carbon metabolism of host cells were commandeered by SARS-CoV-2 to commit to de novo purine synthesis that was required for viral replication [33]. Moreover, this study also evaluated the in vitro action of methotrexate, a widely used antifolate drug, on SARS-CoV-2 replication. Methotrexate significantly reduced sub-genomic RNA level and N protein expression, and blocked virion secretion at the concentration of 1 µM, exhibiting promising antiviral property against SARS-CoV-2 [33]. Meanwhile, a clinical trial is heading to appraise the safety and efficacy of methotrexate in patients diagnosed with mild COVID-19 (NCT04610567).

### Targeting dysregulated kinases of host

Protein kinases are a large family of enzymes with diverse and essential functions within the cell, making them attractive targets in drug development. And many kinases have been found to be involved in viral infection. The phosphoproteomic analyses reveal that SARS-CoV-2 infection facilitates the activation of casein kinase II (CK2) and p38 MAPK, implying a potential role of these kinases in SARS-CoV-2 life cycle [76]. CK2 is a highly conserved serine/threonine kinase ubiquitously expressed in eukaryotic cells, serving essential functions in diverse cellular processes. It has been found to be highly expressed in multiple cancers and exert tumor-promoting effects via the direct interaction with tumor-associated proteins [77, 78]. Due to its oncogenic properties, CK2 inhibitors have been developed as antitumor drugs. Silmitasertib (formerly CX-4945) is a potent, orally bioavailable and selective inhibitor of CK2 and is currently being tested in multiple clinical trials for various cancers. Recently, silmitasertib has been granted Fast Track designation by the FDA as a potential therapeutic option for medulloblastoma. Besides, CK2 has been found to play a role in viral infection. Viruses can employ CK2 for the phosphorylation of viral proteins to sustain replication process [79, 80]. Recent studies indicate a potential link between CK2 and COVID-19. As mentioned above, SARS-CoV-2 infected cells show increased activity of CK2, meanwhile, the interactome analysis suggests a direct interaction of CK2 and N protein of SARS-CoV-2 [56]. An in vitro study further confirmed the antiviral activity of silmitasertib [half maximal inhibitory concentration (IC_50_) = 2.34 μM] [76]. Currently, two phase 2 clinical trials are underway to evaluate the benefits of silmitasertib in patients with COVID-19 (NCT04663737, NCT04668209).

Rapamycin (sirolimus) and rapamycin derivatives are specific allosteric inhibitors of mTOR complex 1 (mTORC1). Rapamycin has been approved for use in organ transplantation and lymphangioleiomyomatosis, while its two analogs, temsirolimus and everolimus, have also been approved for clinical use in multiple cancers. In addition to the antitumor action, they are demonstrated to have potential antiviral activities. The SARS-CoV-2 interactome reveals the interaction between N protein and la-related protein 1 (LARP1) in RNA biology [56]. LARP1 knockdown resulted in a significant elevation of viral RNAs, while LARP1 overexpression caused a reduced abundance of viral RNAs, suggesting a vital role of LARP1 in viral replication [81]. LARP1 is a known translational repressor regulated by mTORC1. Thus, rapamycin is proposed as a candidate drug for COVID-19 [82]. However, a recent study found an increasing SARS-CoV-2 replication level after rapamycin treatment, which was inconsistent with previous study [81]. Consequently, more studies are needed to explore the role of LARP1 and mTOR in SARS-CoV-2 replication before the application of rapamycin in COVID-19.

### Targeting virally encoded proteins

Mpro is indispensable for coronavirus replication, as it is responsible for the cleavage of polyproteins to generate nsps [83]. Previous studies have confirmed that lopinavir and ritonavir are capable of inhibiting the activity of Mpro and they also show exceptional performance in clinical application [83]. Besides, more studies have been performed to screen novel inhibitors of Mpro. Among them, some antitumor drugs exhibit good performance in suppressing Mpro activity. Masitinib is a specific inhibitor of cKIT with potent antitumor activity and being actively tested in clinical trials for cancer therapy. A recent study has shown that masitinib is able to suppress the enzymatic activity of SARS-CoV-2 Mpro in vitro, and X-ray crystallography and biochemical assays further suggest that it is a competitive inhibitor of Mpro [84]. Mice infected with SARS-CoV-2 and then treated with masitinib show reduced viral load, improved lung pathology and better survival prognosis [84]. However, its antiviral activity seems to be independent of its kinase activity, which requires more studies to clarify the detailed mechanism [84]. Masitinib is currently under phase 2 clinical trials for COVID-19 (NCT05047783).

## Drugs modulating immune and inflammatory responses

**Fig. 2:**
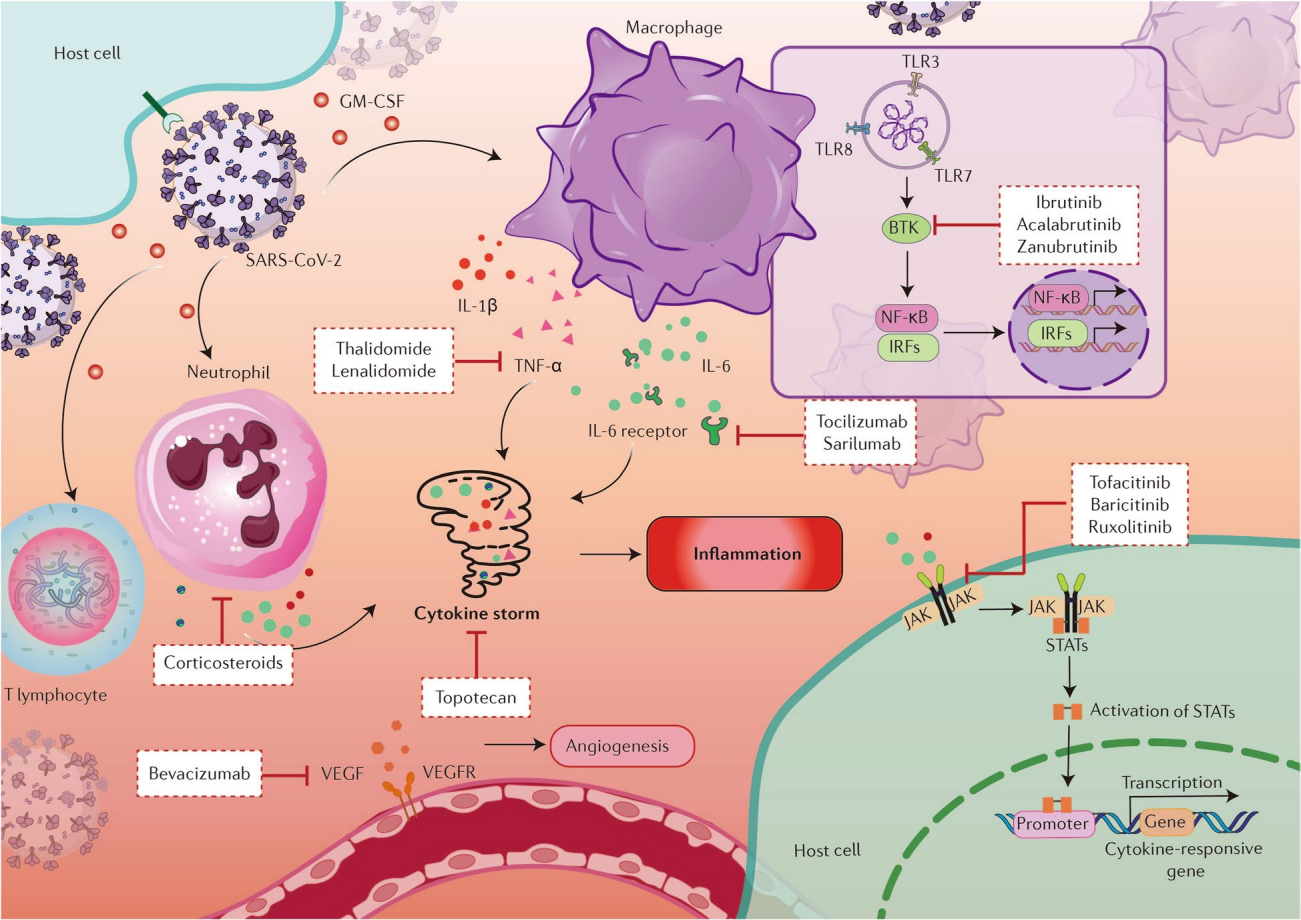
Schematic depiction of hyperinflammatory condition and cytokine storm induced by SARS-CoV-2 infection and potential antitumor drugs to control excessive inflammation. SARS-CoV-2 infection activates macrophages, neutrophils and T lymphocytes. Macrophages and neutrophils produce and release a vast set of pro-inflammatory cytokines such as interleukin-6 (IL-6), tumor necrosis factor alpha (TNF-α), and interleukin-1 beta (IL-1β). These cytokines lead to the activation of JAK-STAT signaling pathway in normal cells and ultimately contribute to the expression of inflammatory genes, further exaggerating the inflammatory responses. Several antitumor drugs target those cytokines and their downstream signaling pathways are considered as potential antiviral agents to control cytokine storm and excessive inflammation. Besides, the upregulation of vascular endothelial growth factor (VEGF) in patients with COVID-19 exacerbates tissue hypoxia. Bevacizumab, one of the anti-angiogenic drugs, blocks the interaction of VEGF-A and its receptor, thus suppressing angiogenesis and increasing tissue perfusion. (Created with the assistance of smart.servier.com)

During SARS-CoV-2 infection, delayed early innate immune response and subsequently exuberant inflammatory response result in viral escape and tissue damage. Thus, strategies to boost the innate immunity and dampen aggressive inflammatory response are likely to effectively clear the virus and avoid tissue damage. Numerous studies have attempted to dissect immunopathological changes of COVID-19 patients which may provide critical resources to identify potential targets for drug discovery. The type I and type III interferons (IFNs) are vital components of innate immunity. Accumulating evidence has demonstrated that delayed and impaired type I and type III IFN responses are common immunological events in patients with severe COVID-19, which are induced by SARS-CoV-2 via its viral proteins as an immune evasion strategy to buy time for viral replication [85–87]. Thus, key molecules involved in IFN response may be potential therapeutic targets for COVID-19. Recently, the cytokines profiles of patients with COVID-19 were analyzed and the results showed significantly higher levels of multiple inflammatory cytokines in COVID-19 patients, especially those at severe progression stage, indicating a correlation between cytokine storm and disease severity [86, 88]. The cytokines with robust increasing levels among COVID-19 patients emerge as attractive targets controlling cytokine storm. These findings, taken together, establish IFN signaling and inflammatory cytokines as promising targets for the management of dysregulated immune responses in severe cases of COVID-19 (Fig. 2).

### Innate immune-activating agents

The innate immune system acts as the frontline defense against invading pathogens, and also plays a critical role in the prevention of tumorigenesis. In mammalian cells, cyclic GMP-AMP synthase (cGAS), a cytosolic DNA sensor, and its downstream signaling effector stimulator of interferon genes (STING), form a major DNA sensing mechanism. The activation of cGAS-STING pathway triggers the production and secretion of type I IFNs which subsequently orchestrate innate immune response [89]. In immuno-oncology, experimental evidence indicates a central role of the host STING pathway as a trigger for spontaneous priming of CD8^+^ T cells against tumor-derived DNA [90, 91]. Also, STING pathway activation leads to enhanced antitumor immune response [92]. Thus, STING agonists have emerged as potential antitumor agents and many of them have advanced to clinical trials.

STING signaling has also been found to play a role in RNA virus infection [93–95]. Recent data from experimental studies have shown that SARS-CoV-2 infection leads to mild activation of STING at later time points of infection, which is required for IFN response against SARS-CoV-2 [96, 97]. STING agonists are thus considered as potential antiviral agents given its role in boosting innate immune response. Dimeric amidobenzimidazole (diABZI) as a highly potent STING agonist has shown strong antitumor activity in a mouse model with colon cancer [98]. Recently, diABZI was demonstrated to be able to boost IFN response, thereby limiting SARS-CoV-2 replication in vitro and in vivo via STING-TANK-binding kinase 1 (TBK1)-IFN regulatory factor 3 (IRF3) signaling pathway [97, 99, 100]. A marked inhibition of SARS-CoV-2 infection was achieved in vitro following treatment with diABZI with a nanomolar EC_50_ and low toxicity, and intranasal administration of 10 μg diABZI protected the mice from SARS-CoV-2 infection via the activation of IFN response [97]. In addition, diABZI also exhibited the same prophylactic and therapeutic effects against SARS-CoV-2 variant B.1.351 (rename Beta) infection with the single 3 μg dose in vivo [97]. These observations suggest that diABZI is a highly potent antiviral agent against SARS-CoV-2. Of course, further clinical testing is needed to evaluate its therapeutic effect in patients with COVID-19.

### Cytokine-neutralizing agents

IL-6 is a member of the IL-6 cytokine family and functions in the regulation of immunity, tissue homeostasis and metabolism. Dysregulation of IL-6 and key downstream signaling pathways are common events in cancer and portend an adverse outcome. IL-6 helps to create an inflammatory and immunosuppressive tumor microenvironment that promotes tumor angiogenesis and outgrowth [101–105]. Thus, targeting IL-6 has been considered as a promising antitumor strategy. Tocilizumab and sarilumab, two monoclonal antibodies against IL-6 receptor (IL-6R), suppress IL-6 signaling pathway and are recommended for the treatment of rheumatoid arthritis and cytokine storm caused by chimeric antigen receptor T (CAR-T) cell therapy in cancer patients.

As a critical mediator of the inflammatory response, IL-6 levels are obviously elevated among COVID-19 patients with complicated disease and are closely associated with adverse clinical outcomes [106]. A recent study demonstrated that lower expression of IL-6, as a result of genetic variant, showed protective effects against critical conditions in patients with COVID-19 [107]. Thus, inhibition of IL-6 pathway may be beneficial to patients with severe COVID-19. Currently, IL-6R blockers are being actively evaluated in COVID-19 clinical trials. However, the results from these clinical trials are inconsistent or even reversed. Some clinical trials have shown that tocilizumab is not effective in preventing poor outcomes and death in hospitalized patients with moderate to severe COVID-19 disease (NCT04356937, NCT04320615), whereas other studies have yielded opposite conclusions about the efficacy of tocilizumab in reducing adverse outcomes but have denied its benefits in improving survival (NCT04372186) [108–110]. There are also some clinical trials demonstrating that both tocilizumab and sarilumab can improve clinical outcomes including survival of patients with severe COVID-19 (NCT02735707) [111]. Moreover, a recent meta-analysis including 27 randomized clinical trials has shown that the administration of IL-6 antagonists is associated with reduced 28-day all-cause mortality of critically ill patients with COVID-19 [112]. The discrepancies among different clinical trials results may be due to the variabilities in disease severity, previous therapeutic interventions and other baseline characteristics of patients enrolled in different studies. The debate about whether IL-6 targeted agents improve clinical outcomes for severe COVID-19 patients will continue, but it also prompts us to think more deeply about the application of cytokine antagonists in COVID-19, such as identifying those who are most likely to benefit from cytokine therapy and the optimal timing for the administration of drugs during the disease course.

### Other immunomodulatory agents

Several cytokines involved in COVID-19 including IL-6 mainly act by Janus kinase-Signal transducer and activator of transcription (JAK-STAT) signaling, one important pathway with a pivotal role in various biological processes including hematopoiesis, immune development, inflammation control, adipogenesis*,* etc. As major downstream effectors of cytokine signaling, JAKs can be therapeutic targets of cytokine-dependent inflammatory and neoplastic disorders. Actually, mutations in the *JAK* family have been observed in hematological malignancies and solid tumors [113–115]. Currently, clinical evaluations of JAK inhibitors (JAKi) in cancer patients are underway.

Recent studies suggest that JAK is involved in exaggerated inflammatory responses triggered by SARS-CoV-2 infection. First, JAK is an important downstream signaling molecule that mediates the proinflammatory effect of IL-6 and other cytokines. Second, IFN signaling activated upon SARS-CoV-2 infection leads to excessive activation of complement system in a JAK/STAT-dependent manner, and ruxolitinib, a JAK1 inhibitor, normalizes the local production of active complement component from infected cells to prevent tissue damage [116]. Remarkably, despite marked efficacy of JAKi monotherapy, combined therapy of JAKi with antiviral agents such as remdesivir shows better curative effect [116]. Several JAKi including tofacitinib, baricitinib and ruxolitinib are being tested in patients with COVID-19. All these three drugs exhibited favorable safety profiles when administered alone or in combination with other antiviral agents via the oral route, and the use of them caused a lower mortality risk among hospitalized patients with COVID-19, most convincingly for baricitinib [117–119]. On November 19, 2020, FDA issued an Emergency Use Authorization (EUA) for baricitinib in combination with remdesivir to treat those hospitalized patients who require ventilatory support [120]. A multinational phase 3 randomized clinical trial further demonstrates its efficacy in improving mortality of critically ill COVID-19 patients (NCT04421027) and multiple phase 3 trials are ongoing [121].

Tumor necrosis factor alpha (TNF-α) is another important inflammatory cytokine that shows increased production in patient with moderate to severe COVID-19. Evidently, TNF-α can trigger robust cell death by activating apoptotic signaling pathway, and blocking TNF-α with neutralizing antibodies in a murine infection model can significantly reduce SARS-CoV-2 induced mortality [24]. Thalidomide and lenalidomide, as anticancer agents, are commonly used to treat multiple myeloma. Both of them can inhibit the production of TNF-α. Several clinical trials are planned or underway to evaluate their efficacy. In addition, other drugs targeting one or more cytokines have also been attempted for COVID-19 treatment but fail to achieve the desired therapeutic effect.

Corticosteroids are known to have anti-inflammatory and anti-allergic effects, and are widely prescribed for the treatment of inflammatory diseases and cancer. Notably, corticosteroids were used to fight against SARS in 2003. However, the administration of corticosteroids in treating infectious diseases has always been the subject of debate. On the one hand, studies reported that the proper use of corticosteroid in critical SARS patients resulted in lower mortality [122]. However, a later study showed that corticosteroid therapy in patients with MERS was not associated with reduced mortality, and even resulted in delayed MERS coronavirus RNA clearance [123]. Despite continuous debate, some critically ill patients indeed benefit from corticosteroids therapy, and the underlying mechanisms are gradually being revealed. For example, a recent study indicates that dexamethasone alters circulating neutrophil states of severe COVID-19 patients by depleting interferon^active^ neutrophils that promote immune response and expanding immunosuppressive immature neutrophils [124]. A large number of clinical trials are being conducted to assess the effectiveness of corticosteroid-based therapy in COVID-19. A prospective meta-analysis reveals a correlation between systemic corticosteroids treatment and lower 28-day all-cause mortality of patients with severe COVID-19 [125]. Thus, the clinical use of corticosteroids should be considered only for those with critical illness, and the dosage and duration must be assessed with great caution.

Pulmonary angiogenesis is one of the distinctive angiocentric features of COVID-19 and shows a strong association with disease severity [126]. The upregulation of proangiogenic factors including vascular endothelial growth factor (VEGF) has been observed in patients with COVID-19 [127]. It is clear that aberrant neovascularization can exacerbate lung tissue hypoxia and inflammation. And blocking angiogenesis will increase tissue perfusion and attenuate inflammation, thus alleviating clinical symptoms of patients. Thus, anti-angiogenesis therapy is a potential strategy that may benefit patients with COVID-19. Bevacizumab is the first available antiangiogenic agent which binds to VEGF-A and thereby blocks the interaction of VEGF-A and its receptor. It has been approved by FDA for the treatment of multiple solid tumors since 2004, and exhibits superior efficacy. A single-arm trial suggested that administration of intravenous bevacizumab improved the oxygenation of patients with severe COVID-19, and, importantly, bevacizumab-related serious adverse events were not observed (NCT04275414) [128]. More randomized controlled trials are warranted to further validate its efficacy in COVID-19.

Topoisomerase 1 (TOP1) is one of the key enzymes involved in the regulation of DNA topology, which binds to duplex DNA and introduces a transient single-strand nick, allowing the rotation of the intact DNA strand around the break, thus resolving topological constraints that impede DNA replication. Once DNA is relaxed, TOP1 religates the nicks. But this religation process can be inhibited by TOP1 inhibitors which trap TOP1 and result in DNA single-strand breaks, eventually leading to cell death [129]. Therefore, TOP1 inhibitors are commonly used as antitumor drugs. Topotecan, one of the TOP1 inhibitors, has been approved by FDA as a second line treatment of metastatic ovarian cancer and small-cell lung cancer. A previous study showed that TOP1 resolved topological constraints of infection-induced genes, leading to the transcriptional activation of pro-inflammatory genes during bacterial and viral infection [130]. As supported, inhibition of TOP1 decreased the expression of pro-inflammatory genes and protected mice from lethal inflammation induced by infection [130]. Recent studies have investigated the effects of topotecan in suppressing excessive inflammatory responses upon SARS-CoV-2 infection. It showed that topotecan had a broad inhibitory action on multiple inflammatory mediators activated by SARS-CoV-2 infection both in vitro and in vivo, and intraperitoneal administration of topotecan at late stage post-infection with 2 mg kg^−1^ body weight dosing markedly improved morbidity outcomes of infected mice without affecting viral clearance kinetics [131]. These results implicate a potential role of topotecan as an anti-inflammatory agent in the treatment of COVID-19. Currently, a clinical trial has been launched to investigate the efficacy of topotecan in relieving hypoxia among patients with COVID-19 (NCT05083000).

Bruton’s tyrosine kinase (BTK) is known as a key constituent of B cell receptor signaling which is critical for the proliferation and survival of both normal B cells and malignant B cells. Thus, BTK is an attractive target for developing therapies to fight against B cell malignancies. To date, BTK inhibitors including ibrutinib, acalabrutinib and zanubrutinib, have been successively approved for patients with lymphoid malignancies, and more inhibitors are under development or investigation in clinical trials [132, 133]. Recent studies have revealed a broader potential role of BTK in other innate immune cells beyond B cells. Macrophages recognize invasive pathogens via their toll-like receptors (TLRs) and trigger inflammatory responses through BTK-dependent activation of nuclear factor kappa-B (NF-κB) [134, 135]. Besides, BTK is also a direct positive regulator of NOD-like receptor thermal protein domain associated protein 3 (NLRP3) inflammasome, contributing to the production and secretion of multiple proinflammatory cytokines [136, 137]. Thus, BTK can be recognized as a positive regulator of inflammation. Interestingly, one study in waldenstrom macroglobulinemia patients with newly diagnosed COVID-19 found that oral ibrutinib at a regular 420 mg d^−1^ dosing improved oxygenation and decreased inflammatory cytokines of patients, implicating a pro-inflammatory role of BTK in COVID-19 [138]. In addition, a recent study reported the specific activation of BTK in monocytes from patients with COVID-19, with a concomitant increase of IL-6 production, and most patients receiving oral acalabrutinib showed rapidly improved oxygenation and clinical status as well as normalized inflammatory mediators [139]. Acalabrutinib is a more specific inhibitor of BTK compared with ibrutinib. In this regard, acalabrutinib is a more ideal candidate for COVID-19 with relatively favorable safety profile, although no severe toxic side effects have been reported in both drugs when used in patients with COVID-19. Currently, several clinical trials are ongoing for further assessment of their performance.

## Challenges and solutions

Despite many advantages, repurposing existing drugs to treat COVID-19 encounters multiple challenges. Firstly, repurposed candidates with established therapeutic efficacy against SARS-CoV-2 in screening models may show limited effects in clinical trials. Currently, drug repurposing screens for COVID-19 are majorly based on transformed cell lines, and the screened drugs are subsequently validated in primary human cell models or animal models. However, these screening models, whether cell models or animal models, fail to entirely recapitulate the physiological conditions of human SARS-CoV-2 infection, which constrains the accurate assessment of the efficacy of drugs to some extent. Thus, the screened repurposed drugs, although exhibiting high efficacy in cell lines and animal models, may fail to achieve the expected treatment response in patients with COVID-19. To overcome the limitations of cell lines, various organoid culture systems which can simulate the process of SARS-CoV-2 infecting different human organs have been, or are being, developed and used to test potential therapies for COVID-19 [140–142]. In addition, more accurate screening models for drug repurposing screen are still needed to be developed and refined.

Secondly, some repurposed drugs tend to have higher therapeutic dosages in COVID-19 treatment than their original indications, which may raise the safety concerns and lead to increased risk of side effects. In this scenario, topical administration is an alternative strategy which enables high local drug concentrations in diseased organ. For example, STING agonist diABZI can achieve therapeutic effect in a transgenic mice model of SARS-CoV-2 infection at the 0.5 mg kg^−1^ body weight dose level when administered intranasally, much lower than its treatment dose 1.5 mg kg^−1^ body weight in mouse model of colon cancer. Thus, nasal administration is a viable strategy for reducing the required dose of repurposed drugs. Besides, developing organ-targeted drug delivery system is another promising approach allowing for lower drug dosing and reduced off-target effects. Indeed, some novel lung-targeted lipid nanoparticle (LNP) delivery systems have been reported to deliver small interfering RNA (siRNA) and mRNA to the lungs successfully [143, 144]. Targeted delivery can be considered for those small molecule drugs with multiple molecular targets which are more likely to cause unwanted side effects.

Thirdly, drug resistance often develops with the emergence of SARS-CoV-2 new variants. Mechanistically, these potential drugs exert antiviral effects either by directly targeting viral components, or by indirectly targeting host cell signaling pathways that viruses are dependent on for entry or replication. Direct-acting antiviral agents (DAA) have little side-effects compared with host-targeting antivirals (HTA) which have broad effects on various body tissues or cells. However, due to the high variability of the RNA viruses including SARS-CoV-2, drug resistance often develops during the administration of DAA. In this regard, targeting host cell pathways and conserved viral components can reduce the occurrence of drug resistance. HTA, like FASN inhibitor TVB-2640 and STING agonist diABZI, have been shown to have antiviral activities against SARS-CoV-2 variants [66, 97]. For SARS-CoV-2, Mpro and RdRp are relatively conserved structures compared with S protein which is prone to mutations [145]. Repurposed drugs targeting these conserved viral proteins can retard the occurrence of drug resistance in COVID-19 treatment. In addition, a combined therapy will provide an ultimate solution to drug resistance, which combines two or more agents that have distinct mechanisms of antiviral actions to minimize drug resistance.

Finally, targeting host factors is a risky therapeutic strategy. Targeting host metabolic pathways is accompanied by a potential risk for normal cell cytotoxicity that can cause antiviral-related side effects. As mentioned above, topical administration and targeted drug delivery can minimize these off-target effects. Targeting hyperactivated host inflammatory pathways not only limits excessive inflammatory responses and attenuates tissue damage, but also compromises antiviral immunity, which may lead to viral spread within host and increases the risk of secondary infections in patients with COVID-19. Thus, accurately estimating the appropriate timing of initiating anti-inflammatory treatments is very critical to achieve desired therapeutic outcomes. The identification of specific biomarkers of hyperinflammatory condition can also help physicians to recognize the disease course and initiate anti-inflammatory therapy timely. Furthermore, more multicenter randomized clinical trials are warranted to provide accurate information about the rational therapeutic timing and therapeutic efficacy of these agents before clinically used for COVID-19.

## Conclusion and outlook

The COVID-19 pandemic remains a serious public health concern globally and more effective therapies are still urgently needed, especially for those with severe COVID-19. Drug repurposing, as an efficient and time-saving strategy, promises more available agents for COVID-19. Some anticancer drugs, for example, have been repurposed to treat COVID-19, based on common pathophysiological features including dysregulated immune responses and aberrant metabolic pathways which usually act as initiators and/or contributors during the occurrence and progression of both COVID-19 and cancer. Besides, high-throughput screening (HTS) technologies greatly expedite the discovery of anticancer agents that show antiviral properties, improving the success rate of drug repurposing.

It is worth noting that drug repurposing is not completely risk-free, and many challenges remain to be addressed. Apart from intellectual property issues, ineffective drug screens caused by imprecise screening models, unrealistic effective dosing, drug resistance of new variants and the obscure effects of immunomodulatory agents are four main challenges as mentioned above. Every challenge lies opportunities and prospects. Optimizing screening strategies and developing organ-targeted drug delivery systems are able to increase the success rate of repurposing drugs for the treatment of COVID-19. Besides, it is necessary to reasonably assess risk–benefit for each individual patient when immunomodulatory agents are under consideration. As for drug resistance of new variants, a combined therapy can be initiated for those suitable patients.

In recent years, proteomic studies and interactome analysis have identified many novel therapeutic targets and drug candidates for SARS-CoV-2; however, some of which have not been validated in COVID-19, such as receptor-interacting protein kinase 1 (RIPK1) targeted by ponatinib, an antitumor drug [56, 76]. Thus, those drug candidates with well-defined cellular targets need to be further investigated in the near future.

## Data Availability

Not applicable.

## Abbreviations

ACE2: Angiotensin-converting enzyme 2
AKT: Protein kinase B
BTK: Bruton's tyrosine kinase
CAR-T: Chimeric antigen receptor T cell
cGAS: Cyclic GMP-AMP synthase
CK2: Casein kinase II
COVID-19: Coronavirus disease 2019
DAA: Direct-acting antiviral agents
diABZI: Dimeric amidobenzimidazole
EC_50_: Half maximal effective concentration
eEF1A2: Eukaryotic translation elongation factor 1 alpha 2
eIF4A: Eukaryotic translation initiation factor 4A
EUA: Emergency Use Authorization
FASN: Fatty acid synthase
FDA: Food and Drug Administration
HCV: Hepatitis C virus
HIV: Human immunodeficiency virus
HTA: Host-targeting antivirals
HTS: High-throughput screening
IC_50_: Half maximal inhibitory concentration
IC_90_: 90% Inhibitory concentration
IFNs: Interferons
IFN-γ: Interferon-γ
IGF1: Insulin-like growth factor 1
IGF1R: Insulin-like growth factor 1 receptor
IL-6: Interleukin-6
IL-6R: Interleukin-6 receptor
IRF3: IFN regulatory factor 3
JAKi: JAK inhibitors
JAK-STAT: Janus kinase-Signal transducer and activator of transcription
LARP1: La-related protein 1
LNP: Lipid nanoparticle
mRNA: Messenger RNA
Mpro: Main protease
mTOR: Mammalian target of rapamycin
mTORC1: Mammalian target of rapamycin complex 1
N protein: Nucleocapsid protein
NF-κB: Nuclear factor kappa-B
NLRP3: NOD-like receptor thermal protein domain associated protein 3
Nsps: Nonstructural proteins
PIKfyve: Phosphatidylinositol 3-phosphate 5-kinase
PI3K: Phosphatidylinositol 3-kinase
PI3P: Phosphatidylinositol 3-phosphate
PI(3,5)P2: Phosphatidylinositol 3,5-bisphosphate
PLpro: Papain-like protease
RARα: Retinoic acid receptor alpha
RARs: Retinoic acid receptors
RBD: Receptor-binding domain
RdRp: RNA-dependent RNA polymerase
RIPK1: Receptor-interacting protein kinase 1
RXRs: Retinoid X receptors
S protein: Spike protein
S-RBD: Receptor-binding domain of spike protein
SARS-CoV: Severe acute respiratory syndrome coronavirus
SARS-CoV-2: Severe acute respiratory syndrome coronavirus 2
siRNA: Small interfering RNA
STING: Signaling effector stimulator of interferon genes
TBK1: TANK-binding kinase 1
Th17: Type 17 helper T cell
TLRs: Toll-like receptors
TMPRSS2: Type 2 transmembrane serine protease
TNF-α: Tumor necrosis factor alpha
TOP1: Topoisomerase 1
VEGF: Vascular endothelial growth factor
ZIKV: Zika virus
